# Acute Multiple Sclerosis Exacerbation After Vaccination With the Johnson & Johnson COVID-19 Vaccine: Novel Presentation and First Documented Case Report

**DOI:** 10.7759/cureus.24017

**Published:** 2022-04-10

**Authors:** Younus Al-Midfai, Winy Kujundzic, Simrun Uppal, Darby Oakes, Sardinas Giezy

**Affiliations:** 1 Internal Medicine, HCA Florida Westside Hospital, Plantation, USA; 2 Internal Medicine, HCA Florida Northwest Hospital, Margate, USA; 3 Internal Medicine, Nova Southeastern University, Dr. Kiran C. Patel College of Osteopathic Medicine, Fort Lauderdale, USA

**Keywords:** covid-19 vaccination, multiple sclerosis relapse, allergy and anaphylaxis, infectious disease control, covid 19, johnson & johnson, multiple sclerosis flare-ups

## Abstract

Multiple sclerosis (MS) is a neurologic disease caused by a chronic autoimmune process resulting in the demyelination of axons within the central nervous system. MS occurs through combined genetic susceptibility and environmental triggers. MS relapses (MSR) are characterized by acute inflammatory reactions and symptoms. Here we present a novel case of MSR following the second dose of the Johnson & Johnson coronavirus disease 2019 (COVID-19) vaccine (Johnson & Johnson, New Brunswick, New Jersey, United States), presenting with paresthesias and left foot deficit. Additional research and studies are necessary to explore the relationship of COVID-19 vaccinations with MSR.

## Introduction

The first defined description of Multiple Sclerosis (MS) in 1868 was by neurologist Jean-Martin Charcot at the Hôpital de Salpétrière in Paris, France. His work differentiated between what would later be known as Parkinson’s disease and MS. Eventually defining Charcot’s triad of MS: intention tremor, nystagmus and scanning speech [1]. MS is a disease characterized by the demyelination of axons within the central nervous system (CNS). It’s a chronic autoimmune and inflammatory process leading to neurological signs and symptoms. Brain imaging in MS patients will include a minimum of two separate lesions within the spinal cord and white matter of the CNS [2].

MS is understood to be a combination of the genetic susceptibility of an individual as well as non-genetic factors that initiate the autoimmune process. MS diagnoses are classified into four different categories, relapsing-remitting MS, secondary progressive MS, primary progressive MS, and progressive relapsing MS [2]. Of all the MS patients, 85% classify as having relapsing-remitting MS, meaning patients experience flare-ups or exacerbations followed by periods of remission. Secondary progressive MS is defined by the worsening of the disease process regardless of periods of remission. Primary progressive MS, however, infers continued decline in the disease process with no periods of exacerbations or remission periods. The rarest of the four classifications is progressive relapsing MS in which the patient experiences relapses but also has progressive decline from the beginning with no periods of remission [2].

MS symptoms are most often identified in patients aged 20-45 years. It’s estimated that 2.8 million people are living with MS worldwide [3]. Neurological deficits can vary, commonly including fatigue, numbness/tingling, and pain. Of the four classifications of MS, the most common form, relapsing-remitting MS, is characterized by episodes of flare-ups of the disease separated by periods of remission [2]. MS relapses (MSR) are defined as acute inflammatory demyelinating reactions within the CNS. Risk factors include age, sex, serum levels of vitamin D, infections, and pregnancy [4]. At this point, it is unclear if vaccines are a risk factor for acute MSR [5].

There is no current documentation of the Johnson & Johnson (J&J) coronavirus disease 2019 (COVID-19) vaccine (Johnson & Johnson, New Brunswick, New Jersey, United States (US)) causing MSR. The J&J vaccine is a recombinant vector vaccine using human adenovirus to express the spike protein of SARS-CoV-2. Phase 3 of the vaccination study was performed in early 2021. The level of protection was determined to be 72% in the US against moderate and severe disease [6]. According to a survey completed by 338,700 individuals the week after receiving their vaccine, it was found that the majority of them experienced systemic and local reactions, and 34% reported a health impact [7].

## Case presentation

A 25-year-old Caucasian female with a medical history of MS presented with dragging of her left foot when ambulating for five days. She reported that her left lower extremity had begun feeling unstable and stiff when stepping down. Over the next few days, there was no change in symptoms. MS clinically isolated syndrome (CIS) had been diagnosed four years before this presentation when the patient had debuted with an episode of optic neuritis that fully resolved with steroid treatment. Magnetic resonance imaging (MRI) of the brain at that time showed findings consistent with MS, and she followed up with a neurologist for a short time but stopped due to not presenting with any further symptoms and was not placed on maintenance therapy. That episode had occurred after she had begun hormonal contraception with intramuscular medroxyprogesterone, and because of that she had held off from continuing any form of hormonal contraceptives, until recently before the current episode when she had decided to restart; she had gotten one shot of Depo-Provera® (Pfizer Inc., New York, US) approximately three months before. In this admission, she reported having received the second dose of the J&J COVID-19 vaccine two weeks prior.

Additional medical history includes a social history of smoking cannabis a few times a week (denied nicotine use), and family history positive for MS in her mother. She had taken the first dose of the J&J COVID-19 vaccine about seven months earlier. She was not taking any medications at home. A pregnancy test was not done. A review of systems was only positive for mild intermittent paresthesias of the left thumb that had started around the same time as her left foot deficit. Vital signs were within normal range, there were no abnormal findings on physical or neurological examination at the bedside. The deficit was only noticeable during gait evaluation, with dragging of the left foot and absent ankle dorsiflexion.

**Figure 1:**
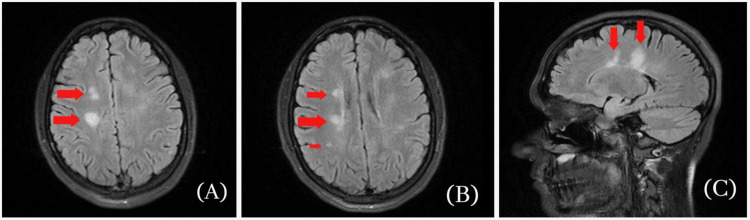
T2-weighted MRI images of the brain. A & B: Axial images of brain MRI demonstrating several foci of FLAIR hyperintensities (red arrows); C: Sagittal image of brain MRI showing large FLAIR in right posterior frontal lobe measuring 1.4 cm (red arrow). FLAIR: fluid attenuated inversion recovery

Laboratory tests (hemogram, comprehensive metabolic panel, urinalysis) were within normal values, computed tomography of the head without the administration of contrast material was unremarkable, and MRI of the brain/spine with contrast showed several foci of fluid-attenuated inversion recovery (FLAIR) hyperintensities (Figures 1), related to the periventricular white matter, the largest one in the right posterior frontal lobe measuring 1.4 cm and consistent with demyelinating disease.

Treatment was started with 1000 mg of IV methylprednisolone daily. Neurology consult advised three total doses of IV Solu-Medrol® (Pfizer Inc., New York, US) and physical therapy. On the third day of hospitalization, the patient reported very mild improvement. Physical therapy provided gait training exercises. She was discharged home with a follow-up neurological appointment in one week.

## Discussion

MS is a chronic autoimmune disorder that damages the CNS. MSR involves an acute inflammatory demyelinating reaction within the CNS, which may be triggered by a multitude of different factors [4]. Research suggests there is a wide range of factors that influence MSR, including age, sex, pregnancy, serum levels of vitamin D, interactions between genetic and environmental factors, and infectious diseases [4]. Many of these factors are modifiable and could potentially aid in MSR prevention and management. The role of viruses in the pathogenesis of MS is presumably related to the activation of self-reactive T-cells that target the CNS; however, the exact mechanism remains unknown [8].

Aside from the yellow fever vaccine, studies thus far have failed to demonstrate a clear relationship between vaccination and MS development or MSR [8]. It has been postulated that the inflammatory response generated by vaccinations in the background of an already altered immune system, such as in those with autoimmune conditions or those susceptible to developing them, may play a role in unmasking the disease after vaccination [8]. Studies have suggested that severe acute respiratory syndrome coronavirus 2 (SARS-CoV-2) vaccines may trigger autoimmune diseases, such as immune-mediated thrombotic thrombocytopenia, CNS demyelinating diseases, inflammatory peripheral neuropathies, myositis, myasthenia gravis, limbic encephalitis, and giant cell arteritis [9]. A study conducted in Italy found that 16 patients who had received vaccination against COVID-19 experienced MSR three days to three weeks following vaccination [10]. Ten patients received the Pfizer-BioNTech vaccine (Pfizer Inc., New York, US; BioNTech SE, Mainz, Germany), two received the Moderna vaccine (Moderna, Inc., Cambridge, Massachusetts, US), and four received the AstraZeneca vaccine (AstraZeneca plc, Cambridge, United Kingdom). Additionally, there have been five reports of newly diagnosed MS following mRNA COVID-19 vaccination; however, the MRI studies demonstrated a mix of old and new lesions, meaning that there may have been clinically latent disease present prior to vaccination [8]. Three of these cases involved vaccination with the Pfizer-BioNTech vaccine and two with the Moderna vaccine. This case report is unique in that the patient was vaccinated with the J&J vaccine two weeks prior to developing the MSR, which has not been documented previously.

## Conclusions

In conclusion, we present a novel case of MSR following COVID-19 vaccination with the J&J vaccine. Such a case has yet to be reported in scientific literature. Although the current data is not significant to establish an association between MSR and COVID-19 vaccination, it is important for clinicians to be aware of the possibility and to monitor MS patients for relapse following vaccination. Advanced preparation for relapse may aid in a more timely diagnosis and treatment of MSR and ultimately better patient outcomes. Additional research is needed to establish an association between MSR and COVID-19 vaccination.
